# Systems issues limiting acute fracture care delivery at a tertiary care hospital in Northern Tanzania

**DOI:** 10.11604/pamj.2024.48.29.41286

**Published:** 2024-05-29

**Authors:** Papa Kwadwo Morgan-Asiedu, George William Fryhofer, William Mack Hardaker, Ajay Premkumar, Max Shin, Sireesh Ramesh, Christian Pean, Mubashir Alavi Jusabani, Rogers Temu, Honest Massawe, Neil Perry Sheth

**Affiliations:** 1 Perelman School of Medicine, University of Pennsylvania, Philadelphia, United States of America,; 2 Department of Orthopaedic Surgery, University of Pennsylvania, Market Street, United States of America,; 3 Department of Orthopaedic Surgery, Duke University Medical Centre, Durham, United States,; 4 Department of Orthopaedic Surgery, Hospital for Special Surgery, New York, United States,; 5 Department of Cardiothoracic Surgery, University of Pennsylvania, Market Street, United States,; 6 Life Sciences and Management Program, University of Pennsylvania, Philadelphia, United States of America; 7 Harvard Orthopaedic Trauma Initiative, Brigham and Women´s Hospital, Massachusetts General Hospital,; 8 Kilimanjaro Christian Medical University College, Sokoine Rd, Moshi, Tanzania,; 9 Department of Orthopaedic Surgery, University of Pennsylvania, Pennsylvania Hospital, Spruce Street, Philadelphia, United States of America

**Keywords:** Orthopaedics, trauma, fracture fixation, fluoroscopy, capacity building, road traffic crash

## Abstract

**Introduction:**

sub-Saharan Africa experiences a significant musculoskeletal trauma burden. Among patients who receive surgical treatment, there have been no reports as to how often surgical care is determined to be “adequate” or, if “inadequate”, then what hospital and orthopaedic specialty-specific systems limitations might be prohibitive.

**Methods:**

data from patients presenting to the orthopaedic trauma service at a tertiary care center in sub-Saharan Africa were prospectively collected over a 6-week period and then retrospectively reviewed to determine whether the surgical treatment was “adequate” (or otherwise, “inadequate”) according to the principle of restoring length, alignment, and rotation. Exclusion criteria included insufficient clinical information; isolated spinal injury; infection; cases involving only removal of hardware; soft-tissue procedures; tumor cases; and medical (non-surgical) conditions.

**Results:**

112 cases were included for analysis. Surgery was indicated in 106 of 112 cases (94.6%), and of those, surgery was performed in 62 cases (58.4%). Among patients who underwent surgery with available post-operative imaging (n=56), surgical treatment was “inadequate” in 24 cases (42.9%). The most common reasons treatment was deemed “inadequate” included unavailability of appropriate implants (n=16), unavailability of intraoperative fluoroscopy (n=10) and incomplete intraoperative evaluation of injury (n=5).

**Conclusion:**

several systems limitations prevent the delivery of adequate surgical treatment in patients with acute orthopaedic traumatic injuries, including lack of intraoperative fluoroscopy and lack of implant availability. This study will serve as a useful baseline for ongoing efforts seeking to improve orthopaedic specialty resource availability and facilitate more effective fracture care in this region.

## Introduction

Sub-Saharan Africa experiences a significant musculoskeletal trauma burden, with road traffic crashes (RTCs) representing a major source of morbidity and mortality [1]. Road traffic crashes often involve the lower extremity and require surgical intervention for definitive fixation. However, due to systems level constraints, appropriate and timely definitive care is not always possible, even at tertiary care facilities. Kilimanjaro Christian Medical Centre (KCMC) is one such tertiary care centre in sub-Saharan Africa that serves a population of 12.5 million people in Northern Tanzania. The baseline burden of orthopaedic disease at KCMC has previously been defined [2], with over 160 fractures presenting during a 2-month study period and more than 95% of patients requiring definitive surgical treatment. Previous studies have shown more broadly how systems level issues can affect patient care in low- and middle-income countries, including lack of transportation to a healthcare facility, limitations in hospital capacity, and the inability of patients to pay for the cost of medical care upon arrival [3].

Given these limitations, only a fraction of patients who require surgery actually receive it, although the proportion of patients that do receive definitive fixation for treatment of orthopaedic injuries remains unknown. Furthermore, even in patients who do receive surgical treatment, there have been no reports as to how often surgical care is determined to be “adequate” or if “inadequate”, then what hospital and orthopaedic specialty-specific systems limitations might be prohibitive. The purpose of this study was to define hospital and orthopaedic specialty-specific systems limitations that prevent the “adequate” delivery of musculoskeletal trauma care among patients presenting to a tertiary care center in sub-Saharan Africa. Data from consecutive patients presenting to the orthopaedic trauma service at KCMC were prospectively collected over a 6-week period and then retrospectively reviewed to determine whether the given treatment was “adequate” (or otherwise, “inadequate”) compared to the general standard of care for a specific fracture pattern. We hypothesized that a significant number of patients that required surgery ultimately would not receive it. Furthermore, we hypothesized that for patients that did receive definitive surgical fracture fixation, treatment would not always be “adequate”, which could be explained by one of several systems limitations.

## Methods

**Study design:** a prospective observational study was performed at the Kilimanjaro Christian Medical Centre (KCMC) in Moshi Tanzania. This involved a six-week period of prospective data collection, followed by retrospective review to determine whether given treatments were adequate or inadequate and evaluation of underlying reasons for inadequate care.

**Setting and time period:** data was prospectively collected for all patients admitted to the orthopaedic surgery ward at KCMC in Moshi, Tanzania from June 18, 2018 - July 31, 2018.

**Participants:** the study included all patients admitted to the orthopaedic surgery ward at KCMC for the specified time period. The majority of patients were admitted to the orthopaedic surgery ward directly from the emergency department. Surgical procedures were performed in a single orthopaedic surgical theater on six out of seven days per week, with one to four procedures performed daily.

**Inclusion criteria:** all patients admitted to the orthopaedic surgery ward at KCMC for the specified time period were included in the study.

**Exclusion criteria:** exclusion criteria included insufficient clinical information (including lack of injury X-rays), isolated spinal injury, infection, cases involving only removal of hardware, soft-tissue procedures, tumor cases (given lack of advanced imaging) and medical (non-surgical) conditions. Infection and tumor cases were excluded because post-hoc review of radiographs alone would not suffice to determine the adequacy of treatment.

**Variables:** for patients included in the study, basic demographics (sex, age), mechanism of injury, fracture location(s), presence or absence of an open fracture, and treatment(s) received were collected. All available X-rays were also obtained for each case and securely stored for further review. External fixation was categorized as definitive surgical fixation, but skeletal traction was not.

**Data sources and measurement:** following the data collection period, hospital records for each case were reviewed by three authors (GWF, WMH, NPS) to determine whether surgery was indicated, with major indications including the presence of an open fracture, length-unstable injuries, gross malreduction (including after attempted closed management), or displaced articular fractures. Final treatment decisions were reviewed to determine whether the surgical treatment provided for each case was thought to be “adequate” within reason according to the principle of restoring length, alignment, and rotation [4] - or otherwise “inadequate” (cases without sufficient post-operative imaging were excluded from this analysis). Additionally, cases of long bone fracture where skeletal traction was used as the definitive form of fixation were deemed “inadequate” by default. Cases with “inadequate” treatment were further sub-classified into one or more reasons for why the treatment was deemed “inadequate”: i) appropriate implant not available; 2) intraoperative fluoroscopy not available; or 3) incomplete intraoperative injury evaluation.

**Ethical approval:** this study received Kilimanjaro Christian Medical Centre (KCMC) Clinical Research Ethical Review Committee approval prior to data collection and analysis.

## Results

**Figure 1:**
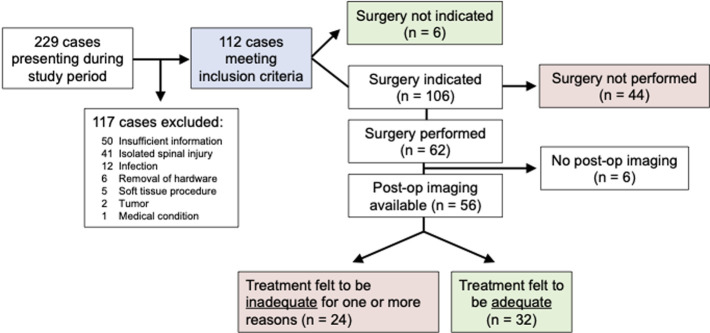
flow diagram of study design and surgical outcomes

**Participants and descriptive data:** a total of 229 cases presented to the orthopaedic service at KCMC during the 6-week study period. Of those, 117 cases were excluded from further study for one of six different reasons (Figure 1). After exclusion, 112 cases remained for analysis. General case information is summarized in Table 1. The study population was 82.1% male (n=92) with a median age of 39.4 years old (range, 2 to 98 years) and a mean of 35.5 years. Mechanisms of injury are summarized in Table 1, with the most common being RTCs (n=58, 52%) and falls (n=40, 36%). Thirty-two of 112 cases (28.6%) included at least one open fracture. Included in the 112 cases were over 180 unique fractures (Table 2). Lower extremity fractures predominated, with the femur (n=60, 33%), tibia (n=24, 13%), and fibula (n=21, 12%) being the most common.

**Table 1:** demographics of patients included in the study and associated mechanisms of injury (n=112)

	n		(%)
**Characteristic**			
Sex	Male	92	(82.1)
	Female	20	(17.9)
Age	>= 50 yrs	25	(22.3)
	< 50 yrs	87	(77.7)
Open fractures	By patient	33	(29.2)
	By fracture	75	(39.3)
**Mechanism of injury**			
Road traffic crash (RTC)		58	(51.8)
Fall		40	(35.7)
Assault		4	(3.6)
Bicycle		2	(1.8)
Construction accident		2	(1.8)
Crush		2	(1.8)
Machete		2	(1.8)
Animal attack		1	(0.9)
Blunt trauma		1	(0.9)
**Total musculoskeletal trauma cases; n = 112**			

**Table 2:** list of injuries by association of osteosynthesis/orthopaedic trauma association fracture classification

Fracture	AO/OTA classification	n	%
**Femur**		**60**	**33.3%**
Diaphyseal, simple	32A	17	28.3%
Trochanteric region	31A	13	21.7%
Femoral neck	31B	12	20.0%
Diaphyseal, wedge	32B	10	16.7%
Diaphyseal, multi-frag	32C	3	5.0%
Complete articular	33A	2	3.3%
Distal extraarticular	33C	2	3.3%
Partial articular	33B	1	1.7%
**Tibia**		24	13.3%
Diaphyseal	42	18	75.0%
Proximal articular	41	4	16.7%
Distal articular	43	2	8.3%
**Fibula**		21	11.7%
Diaphyseal	4F2	17	81.0%
Proximal articular	4F1	4	19.0%
**Foot**		18	10.0%
Metatarsal	87	12	66.7%
Phalanx	88	4	22.2%
Foot crush injury	81	1	5.6%
Talus	89	1	5.6%
**Ankle**		11	6.1%
Supra-syndesmotic fibula	44C	8	72.7%
Trans-syndesmotic fibula	44B	3	27.3%
**Radius**		11	6.1%
Diaphyseal	2R2	6	54.5%
Distal articular	2R3	5	45.5%
**Hand**		9	5.0%
Metacarpal	77	7	77.8%
Phalanx	72	1	11.1%
Scaphoid	78	1	11.1%
**Pelvis**		9	5.0%
Pelvic ring	61	5	55.6%
Acetabulum	62	4	44.4%
**Humerus**		7	3.9%
Distal articular	13	4	57.1%
Proximal articular	11	2	28.6%
Diaphyseal	12	1	14.3%
**Ulna**		7	3.9%
Diaphyseal	2U2	4	57.1%
Distal articular	2U3	3	42.9%
**Other**		3	1.7%
Patella	34	3	100.0%

**Figure 2:**
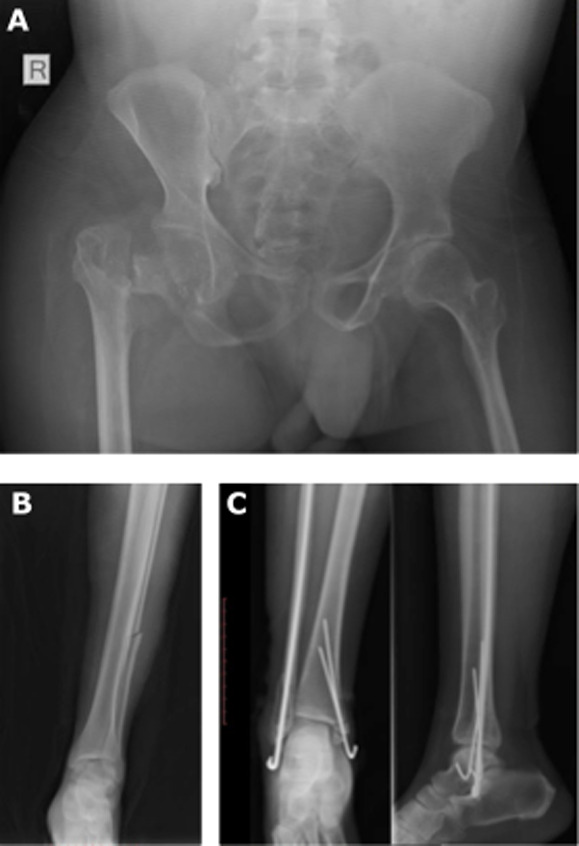
sample patients with inadequate treatment; A) unavailable implants: basicervical femoral neck fracture status-post excisional arthroplasty instead of open reduction internal fixation/total hip arthroplasty; B) incomplete injury evaluation: distal femur/midshaft fibular fracture without appropriate evaluation of ankle and syndesmosis; C) intraoperative fluoroscopy unavailable: postoperative radiographs of bimalleolar fracture demonstrate medial malleolar fragment not captured by k-wire

**Main results and analyses:** surgical intervention was indicated in 106 of 112 cases (94.6%), and of those only 62 cases (58.4%) received surgical intervention. Traction was utilized as definitive treatment in 22 cases (19.6% of all cases), including three tibia fractures, 17 femur fractures, one pelvic ring injury, and one acetabular fracture. Of the patients that underwent surgical fixation, 17 patients (26.6%) received external fixation as the definitive treatment. In 9 of 112 cases (8%), there was either a change from or patient refusal of the initial treatment plan. Patients who underwent surgical treatment were further reviewed to determine the adequacy of the given procedure. Among patients who underwent surgery with available post-operative imaging (n=56), the surgical treatment was deemed “inadequate” in 24 cases (42.9%). Reasons for why treatment was deemed “inadequate” are listed in Table 3 and Figure 2, with “appropriate implant not available” being the most common (n=16), followed by “intraoperative fluoroscopy not available” (n=10) and “incomplete intraoperative evaluation of injury” (n=5). In five cases, more than one system issue was responsible for “inadequate” treatment.

**Table 3:** reasons why treatment was deemed inadequate (n= 24 patients)

Reason(s) why treatment was deemed inadequate	n
Appropriate implant not available	16
Intraoperative fluoroscopy not available	10
Incomplete evaluation of injury	5

## Discussion

Although several studies have examined surgical intervention in developing countries [5-10], few of these have focused on orthopaedics [11], and specifically in sub-Saharan Africa [3,12,13]. This prospective study sought to define hospital- and orthopaedic specialty-specific systems limitations that prevent the “adequate” delivery of musculoskeletal trauma care among patients presenting to a tertiary care center in Tanzania over a six-week period. The basic demographics and injury data observed in this study align with a study performed at a tertiary hospital in Nigeria [14] with a mostly male population, patients on average in the fourth decade of life, and fractures of the femur, tibia, and fibula being the most common injuries sustained as the result of road traffic crashes. Although we observed a high rate of operative injuries (n=106, 94.6%), a significant proportion of cases requiring surgical intervention did not receive surgery (n=44, 41.5%), with patients instead receiving traction and other inadequate interventions. Additionally, in many cases available for post-hoc surgical case review, treatment was deemed “inadequate” (n=24, 42.3%) due to systems limitations including unavailability of surgical implants, unavailable intraoperative fluoroscopy, and incomplete intraoperative evaluation of injury.

These findings raise several important considerations. First, although our assessment of surgical treatment being “inadequate” in over 40% of cases may at first appear discouraging, it must be noted that in all of those cases, “inadequate” care could be explained by one or more potentially correctable systems limitations. Additionally, surgery not being performed in 44 cases in which it was indicated could suggest one of several possible scenarios: i) appropriate implant(s) and/or materials were not available; ii) lack of operating room availability; or iii) patients being unable to pay for the cost of definitive treatment and/or follow-up care. These conclusions are supported by a previous study of orthopaedic surgical treatment delays at a tertiary hospital in Nigeria, which identified lack of operating room availability, followed by lack of funds as two of the most common reasons for operative treatment delays [15]. The previous study also found that weekend admission was also significantly associated with delay in surgery of more than 3 days.

Although not explicitly studied here, there are several limitations in the current system at KCMC that may preclude the delivery of appropriate definitive orthopaedic care: i) lack of operating theater availability; ii) a steady supply of material resources (e.g. orthopaedic implants) needed for definitive fracture fixation may not be readily available; iii) many patients lack the ability to pay for treatment when surgical intervention is required; and iv) the system is forced to rely on junior trainees - the providers often with the least experience - for definitive patient care, due to a lack of a robust workforce, especially when patients present with complex fractures or polytrauma at off-hours. While our findings are context specific, there are many findings of these study that are true of other similar settings with limited resources. One possible solution for addressing the potential shortcomings of orthopaedic care delivery in a trauma center such as KCMC is to improve the availability of orthopaedic specialty resources. Specifically, these findings suggest that the community served by this tertiary care referral center would likely benefit from having the greater resources or funding that would come with an established orthopaedic specialty hospital that could circumvent existing systems limitations. Such benefits might include extended operating room availability, intraoperative fluoroscopy for any case for which it is required, routine availability of post-operative imaging, greater and more reliable orthopaedic-specific implant availability, and a methodology to cross-subsidize surgical costs for patients that otherwise could not afford it.

For example, the Beit CURE International Hospital in Malawi provides a private elective hip and knee arthroplasty service that is used to fund free health care for other patients in need of musculoskeletal treatment [16]. Previous studies have shown that with proper implant availability, simple as well as some more complex fracture morphologies may be successfully managed, even in low-income countries [17,18]. Our study has several strengths. Over a six-week period we prospectively enrolled all patients presenting for orthopaedic trauma care at KCMC, the main tertiary referral hospital in the northern zone of Tanzania, with a catchment area of over 12 million people. Our study setting and patient population increase the generalizability of our findings for this region. Additionally, our prospective enrollment of all patients presenting for orthopaedic trauma care at KCMC minimized the possibility of selection bias. However, our study also has some limitations. First, little to no follow-up information was available for many cases after the initial treatment, and “adequacy” of treatment was purely based on review of X-rays that were available. It is possible that some of these patients with “adequate” X-rays in the immediate post-operative period did subsequently go on to sustain a complication and/or adverse outcome not captured by this study. Limited follow-up is an important problem in the region, which was previously documented in a study of pediatric osteomyelitis performed at KCMC from 2008 to 2010 [19].

This prior study identified poor attendance to follow-up appointments and record-keeping inconsistencies as major challenges to provision of care. Other studies have documented the sub-optimal long-term follow-up care in sub-Saharan Africa [20-22]. Furthermore, an exclusion bias is possible, as patients with higher energy injuries are more likely to receive X-rays due to limited resources. Given that several cases (n=50, 21.8%) initially identified for the study had to be excluded due to insufficient clinical documentation and/or lack of injury X-rays, our final cohort may have an overrepresentation of patients with higher acuity injuries that received X-rays. Similarly, we were also surprised by the high rate of operative injuries in this study, suggesting another possible exclusion bias in the KCMC study population if patients with less severe injuries acceptable for closed treatment elected not to present to KCMC, opting instead for treatment from closer health centers.

## Conclusion

In this prospective study of patients presenting for orthopaedic trauma care at KCMC over a six-week period, we show that the burden of acute orthopaedic trauma in this region is significant, with a high rate of injuries requiring surgical intervention. Unfortunately, many patients receive “inadequate” treatment due to systems limitations including unavailability of appropriate implants, limited operating theatre resources and unavailable fluoroscopy. This study will serve as a useful baseline for ongoing and future efforts seeking to improve orthopaedic specialty resource availability and facilitate more effective fracture care in Northern Tanzania and sub-Saharan Africa.

### What is known about this topic

- *Many parts of sub-Saharan Africa are under resourced and ill-equipped to address the significant trauma burden that can only be addressed through surgical intervention;*
- *The reasons for inadequacy of care are systemic, spanning human resources, medical equipment and supplies, transportation networks and political will;*
- *Systems level interventions are required to adequately scale up surgical care*.

### What this study adds

- *Characterizes the rate of provision of inadequate orthopaedic trauma at a tertiary referral centre in Northern Tanzania;*
- *Highlights specific opportunities for private-public sector partnerships to improve orthopaedic trauma care via provision of orthopaedic implants, intraoperative fluoroscopy and post-operative imaging;*
- *Underscores importance of and barriers to proper follow-up of patients after procedures to ensure adequate care in Northern Tanzania*.
